# The mayfly nymph *Austrophlebioides pusillus* Harker defies common osmoregulatory assumptions

**DOI:** 10.1098/rsos.160520

**Published:** 2017-01-18

**Authors:** Renee Dowse, Carolyn G. Palmer, Kasey Hills, Fraser Torpy, Ben J. Kefford

**Affiliations:** 1 School of Life Sciences, University of Technology, Sydney, Science Building 7, Harris Street, Ultimo, New South Wales 2007, Australia; 2 Unilever Centre for Environmental Water Quality, Institute for Water Research, Rhodes University, Old Geology Building, Artillery Road, Grahamstown, Eastern Cape 6139, South Africa; 3 Institute for Applied Ecology, University of Canberra, Building 3, Canberra, Australian Capital Territory 2601, Australia

**Keywords:** Ephemeroptera, mayfly, osmolality, osmoregulation, salinization

## Abstract

Osmoregulation is a key physiological function, critical for homeostasis. The basic physiological mechanisms of osmoregulation are thought to be well established. However, through a series of experiments exposing the freshwater mayfly nymph *Austrophlebioides pusillus* (Ephemeroptera) to increasing salinities, we present research that challenges the extent of current understanding of the relationship between osmoregulation and mortality. *A. pusillus* had modelled 96 h LC_10_, LC_50_ and LC_99_ of 2.4, 4.8 and 10 g l^−1^ added synthetic marine salt (SMS), respectively. They were strong osmoregulators. At aquarium water osmolality of 256 ± 3.12 mmol kg^−1^ (±s.e.; equivalent to 10 g l^−1^ added SMS), the haemolymph osmolality of *A. pusillus* was a much higher 401 ± 4.18 mmol kg^−1^ (±s.e.). The osmoregulatory capacity of *A. pusillus* did not break down, even at the salinity corresponding to their LC_99_, thus their mortality at this concentration is due to factors other than increased internal osmotic pressure. No freshwater invertebrate has been previously reported as suffering mortality from rises in salinity that are well below the iso-osmotic point. Recently, studies have reported reduced abundance/richness of Ephemeroptera with slightly elevated salinity. Given that salinization is an increasing global threat to freshwaters, there is an urgent need for studies into the osmophysiology of the Ephemeroptera to determine if their loss at locations with slightly elevated salinity is a direct result of external salinity or other, possibly physiological, causes.

## 1. Introduction

Freshwater invertebrates have internal salinity concentrations higher than the environment in which they live, and so have to cope with two main osmoregulatory challenges. First, large volumes of water enter the body. For freshwater insects, this occurs via drinking [1] and/or cuticular permeability [2,3]. Second, from a dilute environment, they need to acquire specific ions at concentrations that support metabolic activity. Osmoregulation is the active control of intra- and extracellular ionic concentrations and volume. It is critical to homeostasis [3,4], and thus the osmoregulatory capacity of aquatic animals places limits on the salinity range they can inhabit [5]. The basics of osmoregulation are thought to be among the best understood physiological processes (see [6,7]).

Aquatic animals are either osmoconformers or osmoregulators. Osmoconforming is easily defined: internal osmotic pressure fluctuates with the external environment, so that both are similar. Animals that osmoconform across all osmotic pressures do not occur in freshwater because their internal environment would be too dilute to support physiological processes [8]. Osmoregulators actively regulate their internal media at a constant osmotic pressure that is different to that of their external environment. There are, however, various complexities to osmoregulation.

Freshwater osmoregulators maintain internal media at a higher osmotic pressure than their external salinity [4]. When these organisms are exposed to increasing salinity, they continue to regulate their internal media. When the external osmotic pressure rises to such an extent that internal and external osmotic pressures are equal, this is termed the iso-osmotic point. Past this point freshwater animals either continue to regulate their internal media at the same osmolality (which will now be less than the external environment) or osmoregulation breaks down [4], and they start to osmoconform. Freshwater animals that osmoconform at salinities above the iso-osmotic point must be capable of tolerating the increased salinity or they will die [8–11].

Critically, at external salinities below the iso-osmotic point, freshwater animals should not suffer adverse effects from increasing salinity because they need to spend less energy on ion regulation. Studies have found increased growth and/or reproduction of freshwater invertebrates [12,13] and fish [14] at intermediate salinities, presumably owing to relatively high osmoregulation costs at low salinities. So osmoregulatory theory would suggest, and the published literature concurs, that freshwater animals should not suffer from an increased external salinity that is below their iso-osmotic point.

Here, we report the findings of a series of experiments to link osmoregulation and mortality in the freshwater mayfly nymph *Austrophlebioides pusillus* Harker (Ephemeroptera: Leptophlebiidae) where findings contrast with the theory described above.

## 2. Material and methods

### 2.1. Field collection, acclimation and general experimental conditions

*Austrophlebioides pusillus* nymphs were collected from the Hunter River at Moonan Flat, New South Wales (NSW), Australia (S 31°55′529^″^ E 151°14′235^″^). Mean salinity measured as electrical conductivity (EC) was 0.211 mS cm^−1^ (±0.085 s.d.), range 0.123–0.314 mS cm^−1^, *n* = 5) at 25°C. Salinity in the Hunter River catchment includes ionic composition similar to that of sea water [15]. In the laboratory, nymphs were randomly allocated to aquaria containing 3 l of aerated copper-free water (Sydney tap water treated with activated carbon filtration, and a 1 µm sediment filter and UV treatment) and were acclimated to 17°C in a temperature-controlled test room for 3 days. *A. pusillus* with wing buds were excluded from all tests. *A. pusillus* were fed crumbled fish flakes up to 24 h prior to experimentation, but were not fed during the experiments. Throughout acclimation and during all experiments, aquaria were checked daily for emergence and mortality. Exuviae and dead nymphs were removed. Aquaria were covered to reduce evaporation. Three series of experiments were conducted, exposing *A. pusillus* to increasing concentrations of synthetic marine salt (SMS; for measured ionic proportions of Ocean Nature, see [16–18]; Ocean Nature, Aquasonic, Wauchope, NSW) as the most common inland saline waters in Australia have ionic proportions similar to sea water [19].

### 2.2. Series 1: mortality

To establish the relationship between salinity and mortality in *A. pusillus*, we conducted three standard 96 h toxicity bioassays where mortality was the response variable. For these tests, *A. pusillus* were directly transferred from the acclimation water to experimental waters. Nymphs were collected over two seasons during March (autumn), September and October (spring). In each of these experiments, the nymphs were exposed to 10 salinity treatments, ranging from 0.001 to 20 g l^−1^ of added SMS (EC = 0.200–28.4 mS cm^−1^), and control treatments with no added SMS. Depending on the seasonal abundance of the field collected nymphs, these experiments used a minimum of 17 and a maximum of 70 *A. pusillus* per aquarium, with a total of 1278 *A. pusillus* used in all experiments. One aquarium was used per treatment and two for the control within each run, with replication provided by repeating the experiment three times. Results of the three temporal replicates were pooled to produce a single dose–response curve. These experiments provided baseline constant salinity dose–response data, and concentrations for subsequent experiments were estimated from this dataset.

### 2.3. Series 2: osmoregulation and direct transfer

The next experiment involved directly transferring *A. pusillus* to aquaria containing 1, 2 and 4 g l^−1^ added SMS, chosen because 10% (at 2 g l^−1^) and 50% (at 4 g l^−1^) of the population incurred mortality over 96 h in the series 1 mortality test. We were also interested in whether or not osmoregulation was affected by lower salinity levels (1 g l^−1^). Nymphs were collected during January (summer). For each treatment, there were three replicate aquaria each containing 100 individual *A. pusillus* nymphs. As a procedural control, *A. pusillus* were also transferred to a replicated control treatment with no added SMS (*n* = 2 aquaria). pH was measured daily and ranged from 7.75 to 7.93 in control aquaria, 7.68 to 7.99 in 1 g l^−1^ SMS, 7.76 to 7.94 in 2 g l^−1^ SMS and 7.83 to 7.98 in 4 g l^−1^ SMS. Osmolality of the aquarium water and the haemolymph of *A. pusillus* were measured. The total number of *A. pusillus* initially allocated to aquaria was 1100, and haemolymph osmolality measurements were obtained using 639 individuals.

### 2.4. Measuring osmolality

Osmotic pressure was measured as osmolality (mmol kg^−1^) using a Vapro 5520 vapour pressure osmometer equipped with a 2 µl sample chamber (Wescor, UT, USA). Osmolality is an expression of the total number of solute particles dissolved in 1 kg of solvent. The osmolality of the external water (hereafter aquarium osmolality) was measured three times per replicate aquarium per exposure period. At each exposure period, five replicate haemolymph osmolality measurements were taken for *A. pusillus* per treatment. On average, two individual *A. pusillus* were required to obtain sufficient volume for a measure of haemolymph osmolality. To extract haemolyph, *A. pusillus* were placed in a plastic weighing tray on ice, to depress rapid movement. Immediately prior to haemolymph extraction, nymphs were quickly dipped in deionized water to remove any external salt, and allowed to move around on lint-free tissue to dry. Individual nymphs were then enclosed in Parafilm (Pechiney Plastic Packaging, Chicago, IL, USA) with pre-punched filter paper (Whatman #1) placed under the middle of the abdominal segments. A thin needle was used to puncture an abdominal segment above the filter paper. Pressure was applied along the side of the nymph to encourage haemolymph bleeding onto the filter paper. Haemolymph osmolality was measured only on surviving nymphs.

### 2.5. Series 3: osmoregulation, mortality and a ramp increase in salinity

Mortality and osmoregulatory effects from gradually increasing salinity (a ‘ramp’ increase) over 72 h were compared with direct transfer effects. The experiment consisted of controls and a treatment replicated three times. Salinity in treatment aquaria was gradually increased from 0 to 10 g l^−1^ SMS (0.194–14.2 mS cm^−1^) over 72 h, and then maintained at this concentration for a further 48 h. Salinity was gradually increased, using a peristaltic pump to pump in saline water; the water exchange rate was calculated using the formula number 3 from Kraul *et al.* [20]. Water was homogenized within the aquaria by situating the incoming saline water line next to the aeration stone. A data logger was used to record EC every 10 min. pH was measured daily and ranged from 7.96 to 8.03 in the treatment aquaria. Each replicate aquarium contained 111 ± 3 *A. pusillus*.

### 2.6. Statistical analysis

The dose–response curve generated from the series 1 experiments was analysed using logistic regression (SPSS Statistics 17.0) to estimate the concentrations lethal to 10%, 50% and 99% of the population (LC_10_, LC_50_, LC_99_). For the series 2 experiments, two factor analysis of variance (ANOVA) was used to compare treatments (SPSS Statistics 17.0), using the data variables haemolymph osmolality, treatment and time (because these experiments were destructive, repeated measures ANOVA was inappropriate). Where there were significant interactions, multiple comparisons were done within levels of a factor using Tukey simultaneous tests (Minitab 15).

## 3. Results

### 3.1. Series 1: mortality

**Figure 1.**
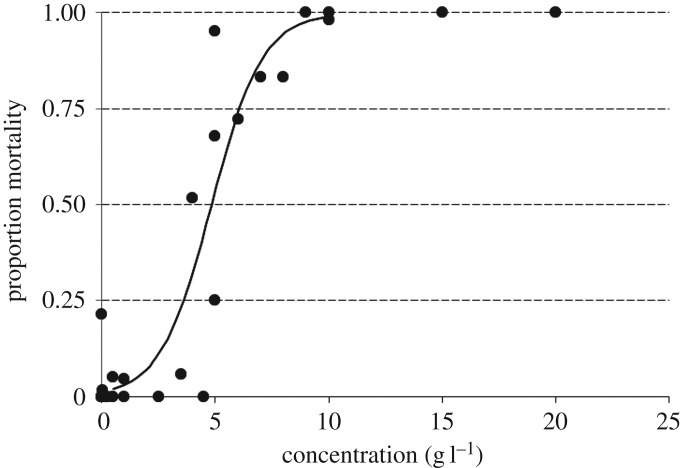
Dose–response curve for the mayfly nymph *Austrophlebioides pusillus* exposed to increasing salinity for 96 h.

At 96 h exposure, there was negligible mortality at salinity concentrations below 2 g l^−1^ SMS (figure 1). At the same exposure period, near complete population mortality occurred at a salinity of 10 g l^−1^ SMS (table 1).

**Table 1.** Modelled salinity concentrations (95% confidence limits) lethal to the mayfly nymph *Austrophlebioides pusillus* (LC*_x_*). Salinity given as g l^−1^ added SMS.

time (h)	LC_10_	LC_50_	LC_99_
72	3.2 (2.2–3.9)	6.5 (5.8–7.2)	12 (11–14)
96	2.4 (1.4–3.1)	4.8 (4.2–5.5)	10 (8.6–12)

### 3.2. Series 2: osmoregulation and direct transfer

**Figure 2.**
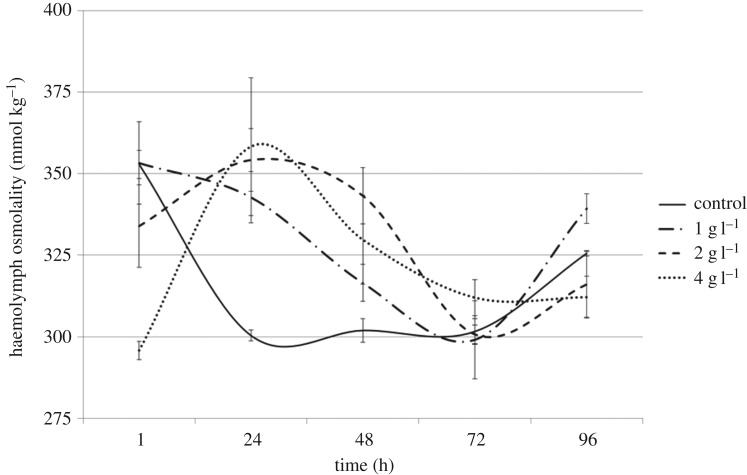
Temporal variability in mean haemolymph osmolality (±s.e.m., *n* = 3) of the mayfly nymph *Austrophlebioides pusillus* at constant salinities of 1, 2 and 4 g l^−1^ added SMS.

*Austrophlebioides pusillus* directly transferred to 4 g l^−1^ SMS maintained haemolymph osmotic pressure within the range of those in the control treatment (table 2), despite this salinity leading to 52% mortality (figure 1). After 96 h exposure, mean haemolymph osmolality of control *A. pusillus* was 326 mmol kg^−1^ (±s.e. 0.80), with a corresponding aquarium osmolality of 11 mmol kg^−1^ (±s.e. 1.30). At the same exposure period, haemolymph osmolality of *A. pusillus* in 4 g l^−1^ SMS was 312 mmol kg^−1^ (±s.e. 6.48) (figure 2), despite the aquarium osmolality of 4 g l^−1^ being almost an order of magnitude higher than that of the control (104 mmol kg^−1^, ±s.e. 2.23).

**Table 2.** Haemolymph and aquaria osmolality (mmol kg^−1^) and salinity (measured as electrical conductivity (mS cm^−1^ at 25°C) pooled for all exposure periods, during direct transfer experiments exposing *Austrophlebioides pusillus* to elevated salinity.

		control (*n* = 2)	1 g l^−1^ (*n* = 3)	2 g l^−1^ (*n* = 3)	4 g l^−1^ (n = 3)
haemolymph	mean (±s.e.)	316 (±6.91)	330 (±6.28)	330 (±6.21)	323 (±7.53)
	range	298–357	284–378	297–364	293–385
aquaria	mean (±s.e.)	8.5 (±1.0)	29 (±0.9)	54 (±1.0)	104 (±0.7)
EC	mean (±s.d.)	0.26 (±0.002)	1.90 (±0.032)	3.42 (±0.053)	6.40 (±0.121)

**Figure 3.**
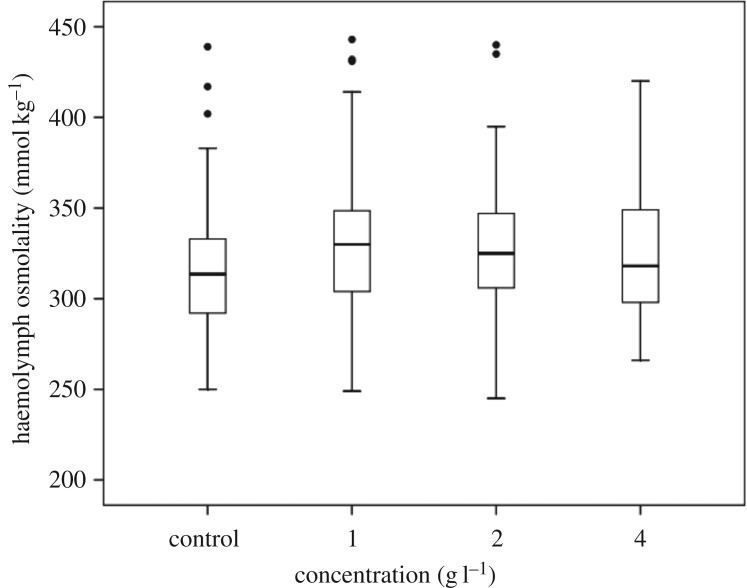
Osmoregulation of the mayfly nymph *Austrophlebioides pusillus* at constant salinities of 1, 2 and 4 g l^−1^ added SMS. Salinity is measured as osmolality. Box = 25th to 75th percentile. Whiskers, minimum and maximum values; central line, median; dot, outlier.

The range of haemolymph osmolality was similar across all treatments including the control (table 2 and figure 3), and there were no detectable differences when the haemolymph osmolality was pooled by treatment across all time points (*p* = 0.396). There was a statistically significant interaction between time and treatment (*p* = 0.002). However, out of a total of 190 multiple (pairwise) comparisons, only eight were statistically different, and there were no consistent patterns between time and treatment. Importantly, despite this interaction, haemolymph osmolality was always much greater than aquarium osmolality (table 2).

### 3.3. Series 3: osmoregulation, mortality and a ramp increase in salinity

**Figure 4.**
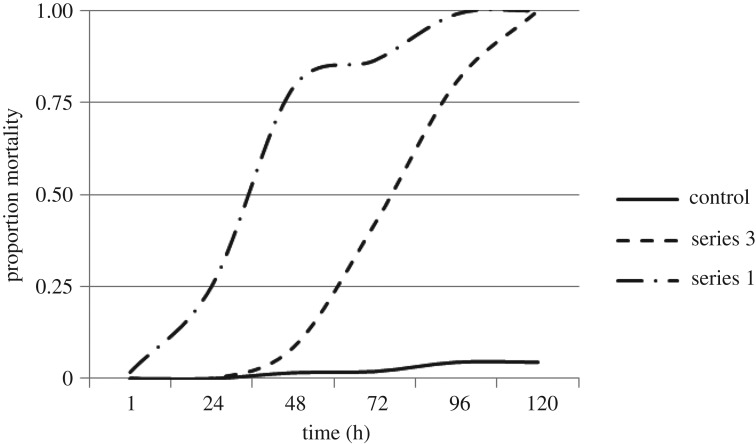
Comparison of mortality for *Austrophlebioides pusillus* under direct transfer (series 1) and ramp increase (series 3) salinity exposures. Series 1 = *A. pusillus* in aquarium salinity of 10 g l^−1^ SMS. Series 3 =* A. pusillus* as aquarium salinity is gradually increased to 10 g l^−1^ SMS over a 72 h period and maintained at this concentration for a further 48 h. Data are means ± s.e.m., *n* = 3.

When salinity was ramped up to 10 g l^−1^ SMS over a period of 72 h, mortality was halved (42% mortality) compared with mortality in the series 1 experiments (86% mortality at 10 g l^−1^ SMS after 72 h; figure 4). However, when 10 g l^−1^ SMS was maintained (after the completion of the ramp increase), mortality increased to 81% after a further 24 h, and 100% after an additional 24 h (or 120 h since the start of the ramp; figure 4).

**Figure 5.**
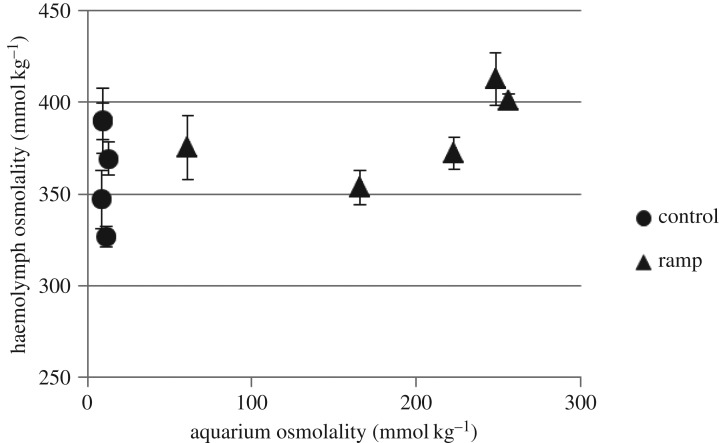
Comparison of mean osmoregulation (measured as osmolality, ±s.e.m., *n* = 3) for *Austrophlebioides pusillus* under control conditions and as aquarium salinity is gradually increased to 10 g l^−1^ SMS over a 72 h period and maintained at this concentration for a further 24 h.

*Austrophlebioides pusillus* exposed to a ramp increase in salinity maintained similar haemolymph osmolality to that of the control animals (figure 5). At 72 h exposure to a ramp increase in salinity, mean haemolymph osmolality was 413 mmol kg^−1^ (±s.e. 14.1, *n* = 3) whereas the mean aquaria salinity was 248 mmol kg^−1^ (±s.e. 3.02, *n* = 3). The corresponding mean haemolymph osmolality for the control was similar (390 mmol kg^−1^ ± s.e. 17.7, *n* = 3) despite the much lower osmolality of the control water (9.33 mmol kg^−1^ ± s.e. 0.33, *n* = 3). Results after salinity was maintained at 10 g l^−1^ SMS for 24 h were similar to those at the completion of the ramp: *A. pusillus* was strongly osmoregulating with the aquarium osmolality at 10 g l^−1^ (256 mmol kg^−1^ ± s.e. 3.12, *n* = 3), which was still much lower than the haemolymph osmolality of *A. pusillus* in this treatment (401 mmol kg^−1^ ± s.e. 4.18, *n* = 3). No *A. pusillus* survived 48 h after the ramp at 10 g l^−1^ SMS.

## 4. Discussion

Ephemeroptera are typically known for their salinity sensitivity under laboratory conditions relative to other stream macroinvertebrates, including acute and chronic exposures to artificial sea water [21–23], and exposures to other salts [24]. The 72 h LC_50_ of 6.5 g l^−1^ SMS obtained for *A. pusillus* in this study corresponds to the only other published 72 h LC_50_ for *Austrophlebioides* spp.: 6.9 g l^−1^ [25]. Furthermore, recently field studies have shown loss of Ephemeropteran or combined Ephemeroptera, Plecoptera and Trichoptera (EPT taxa) richness or abundance at surprisingly low salinities, ranging from 0.1 to 0.7 g l^−1^ (or 0.149–0.997 mS cm^−1^) [26–28]. While these field studies did not measure haemolymph osmolality, the haemolymph of aquatic insects is in the range of 250–400 mmol kg^−1^ [29] which exceeds 10 g l^−1^, with an ionic composition similar to sea water. Thus, Ephemeroptera or EPT taxa are being lost at salinity levels corresponding to an order of magnitude lower than the osmolality of their haemolymph.

Salinity-induced mortality in *A. pusillus* nymphs was not related to a breakdown in osmoregulatory capacity. *A. pusillus* were able to osmoregulate in salinities that were associated with near complete population mortality. They are strong regulators and it is clear that there is no increase in haemolymph osmolality relative to increases in aquaria osmolality. *A. pusillus* may have experienced changes in the internal concentrations of particular ions, internal pH or membrane integrity without the breakdown of osmoregulation. In the absence of data on such phenomena, we will not speculate further.

There were some minor but statistically significant interactions between haemolymph osmolality and exposure time. However, only eight out of a total of 190 multiple pairwise comparisons were statistically different, and there were no consistent patterns between treatment and time. These results are thus consistent with random (type 1) errors. The temporal variability in haemolymph osmolality seen in some treatments was minimal, and showed no evidence for a breakdown in osmoregulation with the changes in internal osmolality not mirroring changes in the external environment.

The biological relevance of the statistically significant interaction between treatment and exposure period is negated by the fact that this species could not withstand environmental salinities nearing internal osmolality. The haemolymph osmolality of *A. pusillus* was always higher than (or hyperosmotic to) aquarium osmolality, and we made no observations of haemolymph osmolality which suggested that osmoregulation had ceased; even at 10 g l^−1^ SMS (the 96 h LC_99_), the osmolality of the aquarium was still less than that of the haemolymph. This leads us to conclude that a breakdown in osmoregulatory capacity resulting from high salinity is not the reason for mortality in the nymphs of *A. pusillus*.

This conclusion is surprising, because mortality and osmoregulatory breakdown have been clearly linked, and causality is regarded as a long standing principle, and in no other species has substantial salinity-induced mortality been observed well below the iso-osmotic point. For example, the plecopteran *Paragnetina media* incurred no mortality and osmoregulated up to the iso-osmotic point (297 mmol l^−1^), incurring high mortality beyond this point [30]. At salinities that cause high mortality, haemolymph salinity was only slightly hyperosmotic to the medium. Likewise for the (salinity tolerant) ephemeropteran *Hexagenia limbata*, osmolality increased with increasing salinity up to the iso-osmotic point (8 g l^−1^) [31]. *H. limbata* lost the ability to osmoregulate, began to osmoconform and died under the increasing salinity exposures. Although a breakdown in osmoregulation was evident for both of the above species, *H. limbata* was the only species where the true isotonicity occurred.

Generally, haemolymph osmotic pressure increases with increasing external salinity, but remains slightly hyperosmotic to the external salinity [32,33]. However, there are differences in osmoregulatory strategies among freshwater insects. For example, Wigglesworth [34] showed stages of osmoregulation breakdown for two freshwater mosquitoes, *Aedes aegypti* and *Culex pipiens* (Diptera). For both these species, osmotic pressure remained constant up to a threshold at which osmoregulation broke down, and the animals began to osmocomform (although their haemolymph always remained slightly hypertonic to the medium). At higher salinity concentrations haemolymph chloride levels rose, and the nymphs died. Likewise, in the nymph *Sialis lutaria* (Megaloptera), there was no change in haemolymph osmotic pressure, rather osmoregulation was affected by increasing salinity via an increase in haemolymph chloride levels [35]. Investigations on seven species of dipteran larva found diversity in ion regulation between species, and evidence of phenotypic plasticity and differences in ion uptake within different populations of the same species at extremely low salinities (e.g. 6–8 mmol l^−1^ NaCl) [36,37]. While we did not measure individual ions, mortality could have been caused by a change in the regulation of single ions [33,38–40].

Below the iso-osmotic point, osmoregulatory theory suggests that freshwater animals should not be disadvantaged by increased salinity. Indeed, they may be at an advantage because they need to spend less energy on osmoregulation [12–14]. Patrick *et al*. [30], however, found that *Ae. aegypti,* a strongly osmoregulating freshwater mosquito, maintained constant uptake of Na^+^ and Cl^−^ over some of the salinity range. Similarly, sodium uptake in the mayfly *Maccaffertium* sp. increased with increasing external sodium concentration, despite constant sodium body burdens [41]. These studies suggested that this would be a result of high influx and efflux of the external medium, and therefore this could be energetically costly in mediums of increasing salinity (but below the iso-osmotic point). Irrespective of differences in osmoregulatory strategies and theories on energy expenditure, no freshwater invertebrate has been previously reported to suffer mortality from rises in salinity well below the iso-osmotic point.

The osmoregulation of crustaceans and dipterans, both of which are generally salinity tolerant orders relative to EPT [23], has been extensively studied. Studies on the osmoregulation of salinity sensitive taxa, such as EPT, are much rarer. Furthermore, species with high haemolymph osmolality tend also to be the species that can adapt to elevated salinity [8]. In this study, the haemolymph osmolality of *A. pusillus* exceeded the upper range of 400 mmol kg^−1^ reported for freshwater insects [29], but we show this species to be very salt sensitive: 72 h LC_50_ of 6.5 g l^−1^ SMS compared with a mean 72 h LC_50_ of 38 g l^−1^, *n* = 377 species [23]. We do not know whether the osmoregulatory–mortality response of *A. pusillus* is common in other species. Given the rarity of studies of EPT species [40], the possibility of such a response occurring in other such species cannot be excluded, particularly for animals that have a large surface area for ion and water exchange and the dissolved oxygen breathers [42,43].

Anthropogenic or secondary salinization of freshwaters results from a range of sources including agriculture, mining and from climate change, and is a growing concern throughout the world [10]; EPT taxa appear to be particularly at risk from salinization. Although there is scant information on their osmoregulation [40], consideration of the salinity at which their richness or abundance declines, cf 0.1–0.7 g l^−1^ [26–28], in the field suggests that they are declining below their likely iso-osmotic point. To establish whether these population declines in the field are directly caused by salinity, there is an urgent need for further studies on the osmoregulation of other apparently salt-sensitive EPT species.

## 5. Conclusion

Salinity causes mortality in the mayfly *A. pusillus*, but a breakdown in osmoregulation does not precede death. Furthermore, mortality of 99% of the population occurred at an external salinity which was considerably less than the haemolymph osmolality. Although research has demonstrated a diversity of osmoregulatory responses to increasing salinity, the results of our study challenge the extent of our understanding of the relationship between osmoregulation and mortality in freshwater invertebrates.

## Data accessibility

Data are available from the Dryad Digital Repository: http://dx.doi.org/10.5061/dryad.dk34h [44].
